# A Novel, Sensitive Assay for Behavioral Defects in Parkinson's Disease Model *Drosophila*

**DOI:** 10.1155/2012/697564

**Published:** 2012-07-25

**Authors:** Ronit Shaltiel-Karyo, Dan Davidi, Yotam Menuchin, Moran Frenkel-Pinter, Mira Marcus-Kalish, John Ringo, Ehud Gazit, Daniel Segal

**Affiliations:** ^1^Department of Molecular Microbiology and Biotechnology, Tel Aviv University, 69978 Tel Aviv, Israel; ^2^Interdisciplinary Center for Technology Analysis & Forecasting (ICTAF), Tel Aviv University, 69978 Tel Aviv, Israel; ^3^School of Biology, The University of Maine, Orono, ME 04469, USA; ^4^Sagol School of Neurosciences, Tel Aviv University, 69978 Tel Aviv, Israel

## Abstract

Parkinson's disease is a common neurodegenerative disorder with the pathology of **α**-synuclein aggregation in Lewy bodies. Currently, there is no available therapy that arrests the progression of the disease. Therefore, the need of animal models to follow **α**-synuclein aggregation is crucial. *Drosophila melanogaster* has been researched extensively as a good genetic model for the disease, with a cognitive phenotype of defective climbing ability. The assay for climbing ability has been demonstrated as an effective tool for screening new therapeutic agents for Parkinson's disease. However, due to the assay's many limitations, there is a clear need to develop a better behavioral test. Courtship, a stereotyped, ritualized behavior of *Drosophila*, involves complex motor and sensory functions in both sexes, which are controlled by large number of neurons; hence, behavior observed during courtship should be sensitive to disease processes in the nervous system. We used a series of traits commonly observed in courtship and an additional behavioral trait—nonsexual encounters—and analyzed them using a data mining tool. We found defective behavior of the Parkinson's model male flies that were tested with virgin females, visible at a much younger age than the climbing defects. We conclude that this is an improved behavioral assay for Parkinson's model flies.

## 1. Introduction

Parkinson's disease (PD) is a common progressive neurodegenerative disorder, characterized by the deposition of amyloid fibrils in Lewy bodies (LBs) in the *substantia nigra* pars compacta, leading to loss of dopaminergic (DA) neurons and to severe motor symptoms [1–4]. *α*-synuclein (*α*-syn), a 140-amino-acid protein, is the main component of the LB [5, 6]. Since the aggregation of the protein in the brain has been implicated as a vital step in the development of PD, one path for the current search for drugs is focused on arresting or modifying the pattern of *α*-syn deposition in the brain [7].

With no currently available drug that arrests or slows down the progression of the disease, therapy treats motor dysfunction, and its effectiveness declines as the disease progresses [8]. Therefore, the development and characterization of animal models may hold promise for screening and testing of new drugs that target the pathogenic process itself rather than the symptoms of PD [9].

The fruit fly *Drosophila melanogaster* is a useful organism for studying mechanisms of human neurodegenerative diseases, including PD [10]. Notwithstanding the conspicuous differences between *Drosophila* and humans, many genes and signaling pathways are conserved between them [11]. Feany and Bender [12] reported a *Drosophila* model for PD that expresses normal human *α*-syn, as well as strains expressing each of the two mutant proteins (A30P and A53T) associated with familial Parkinson's disease in all *Drosophila* neurons; the *Drosophila* genome does not contain a clear *α*-syn homolog [12]. Transgenic flies expressing *α*-syn panneurally display aggregates, suffer loss of DA neurons, and exhibit locomotor defects [12]. They are valuable for drug screening and testing [13] as well for identifying genes and cellular processes relevant to PD pathogenesis, many of which are evolutionarily conserved [11].

A few phenotypes have been detected in PD fly models [11] but only three common phenotypes in flies expressing *α*-syn [12]. The first two are (i) accumulation of *α*-syn aggregates, detected with anti-*α*-syn antibody, and (ii) loss of DA neurons in the brain, detected with anti-TH antibody, which specifically recognizes these neurons in paraffin sections or whole-mount brains [12, 14–16]. The third phenotype is a behavioral outcome of nervous system dysfunction, age-dependent defects in locomotion. The latter is clearly deduced using a simple assay: while normal flies climb up a vertical tube (geotaxis), *α*-syn-expressing flies tend to remain at the bottom [12, 13]. Compared to wild-type flies, the transgenic flies with panneural expression of *α*-syn develop locomotor dysfunction at a relatively early age. This behavioral phenotype has been demonstrated in the past by our group and others as an effective tool for screening of new therapeutics for PD [12, 13, 17, 18].

Courtship in *Drosophila* is a stereotyped, ritualized behavior, which requires males to be athletic and to respond rapidly and appropriately to females. This activity accounts for most interactions between adult individuals and is rich in content, complex in structure, and robust in execution [19]. A male fly can perform the entire courtship ritual immediately upon encountering a virgin female, even if he was raised in complete isolation from egg to adult [20]. In these complex actions, the male and female nervous systems function to generate sexual behavior. Male courtship consists of visual, chemosensory, auditory, and mechanosensory signals [21]. The courtship ritual involves orientation of the male towards the female, serenading the female with a species-specific love song (wing vibration), licking the female's genitalia, and attempting copulation [21, 22].

Since courtship involves many neural and motor elements [23], it might be affected by the expression of *α*-syn. Here we demonstrate an overall decline in the behavioral responses of male transgenic flies expressing A30P *α*-syn in the brain [12], when these males are paired with virgin females. This may potentially serve as a novel, more sensitive assay to study locomotor deficits in *Drosophila*. 

## 2. Methods

### 2.1. Strains and Rearing

We used three strains of *Drosophila*: elav-Gal4, UAS-*α*-syn A30P, and Oregon-R (wild type). Flies were reared on standard cornmeal-molasses medium at 25°C. Crosses were conducted using virgin females collected no more than eight hours after eclosion at 25°C or 18 hours after eclosion at 18°C. Crosses were performed at 29°C. Adult offspring (*F*_1_) from the crosses were collected up to 9 days after the beginning of their eclosion at 25°C in order to avoid offspring from the next generation (*F*_2_).

### 2.2. Crosses

Female flies carrying the driver *elav-Gal4* on their X chromosome were crossed to males carrying the *UAS*-regulated *α*-syn A30P transgene located on chromosome 2 (kindly provided by Professor Mel Feany). All *F*_1_ offspring expressed *α*-syn A30P in the nervous system, giving us a model for PD.

### 2.3. Locomotor (Climbing) Assay

5 vials, each containing 10 flies expressing *α*-syn A30P or 10 Oregon-R flies were analyzed for locomotor behavior. The vials were tapped gently on the table and left standing for 18 seconds. The number of flies that climbed at least one cm was recorded. Altogether, we used 100 flies, half expressing *α*-syn A30P and half wild-type Oregon flies.

### 2.4. Courtship Assay

Flies used for the courtship assay were kept in an opaque box. Taking into account the diel periodicity of *Drosophila* courtship behavior [24], the courtship assay was conducted between 9 : 00 and 15 : 00. A 6-day-old virgin male (Oregon-R or *α*-syn A30P) was placed with a 6-day-old Oregon-R virgin female in a cylindrical transparent chamber (*r* = 1.5 cm, *h* = 0.5 cm) for 10 minutes or until copulation occurred. The interaction between the flies was recorded via a digital microscope and later analyzed for sexual activity using a newly developed software termed “*Drosophila* Analysis,” which allows counting the number of times a fly engages in each element and recording the time it spends in each bout of behavior. We recorded courtship of 56 couples; 28 *α*-syn A30P males and 28 Oregon-R males. The following essential features of male courtship were measured: (i) orientation, (ii) wing vibration, (iii) licking, and (iv) attempted copulation; we also recorded the occurrence of copulation [25]. In addition, we recorded a novel behavioral parameter, nonsexual encounters (NSEs), encounters between the male and female flies that did not lead to sexual activity. NSE is a measure of general activity. To measure the responsiveness to the female more directly, we computed a “sexual focus index” (SFI): SFI = 1/(NSE + 1).

### 2.5. Climbing Assay

Currently, the only behavioral assay of PD model flies is the climbing test. Therefore, we performed this test as well. Transgenic flies expressing the mutated **α*-syn A30P *in their nervous system were used for the experiment. The climbing ability of the flies was monitored twice, at day 6 (when the flies were six days old) and at day 21.

### 2.6. Statistical Analysis

Data were categorized as 9 parameters for each fly including “health condition” as a Boolean parameter [26]. We used association rule learning algorithm, which reveals all the “if then” rules that meet the user predetermined thresholds, to determine how the values of the “health condition” field (the dependent variable) are affected by the values of other fields. The analysis was done using a data-mining tool WizWhy [27] to identify the underlying rules that explain the dependent variable—the health condition. WizWhy reveals all positive and negative “if then” rules in the data and a set of necessary and sufficient conditions (“if and only if” rules). Furthermore the algorithm identified, based on the extracted rules, the unexpected cases deviating from the rules and issued predictions for new cases. The rules summary is shown in Table 1.

## 3. Results

### 3.1. Courtship Assay

**Figure 1:**
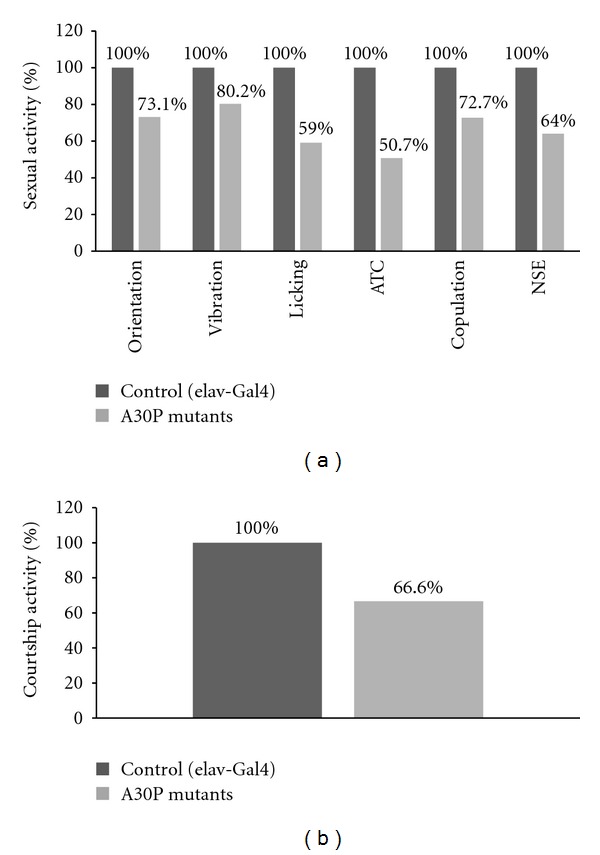
Sexual activity of *Drosophila* flies. Control flies (shown in dark grey) are set as displaying 100% sexual activity. PD model flies are shown in light gray. (a) Courtship behavior was measured using 5 common parameters: orientation, vibration, licking, attempted copulations (ATC), and copulations. In addition, the number of nonsexual encounters (NSEs) was recorded. All parameters were normalized to introduce same scale. (b) Final courtship score, representing the sum of all above 6 parameters.

Sexual activity was normalized to consider Oregon-R flies behavior as ideal (100% sexual activity). Since several couples copulated in less than 10 minutes, we normalized the 4 following measured parameters for each couple, by dividing it in the total time, emphasizing fast copulating males. Orientation time revealed an average of 56.6% in Oregon-R and 41.3% in *α*-syn A30P presenting a 73% orientation activity of mutant flies relative to Oregon-R (41.3/56.6*100). Similarly, Oregon-R vibration time was 21.5% and *α*-syn A30P vibrated for 17.2% of the total time with 80% normal activity. Licking and “attempted copulation” (times 100 and divided by total time) values were 3.4 and 0.4 for Oregon-R and mutant flies, respectively. Thus, *α*-syn A30P flies exhibited a 59% licking activity and 50.7% attempted copulations (ATCs) activity relative to Oregon-R flies. Since copulation is binary, we did not calculate the average copulations per experiment but summed the total copulations that occurred in 28 Oregon-R flies compared to 28 *α*-syn A30P flies. The sum of copulation was 11 copulations in the Oregon-R flies and 8 copulations in the *α*-syn A30P, resulting in 27.3% fewer copulations in PD flies. Interestingly, the two groups differed in nonsexual encounters (NSEs). In A30P *α*-syn flies, mean NSE was 33, and in Oregon-R flies, mean NSE was 21. Here we noticed the occurrence of NSE mainly prior to beginning of any sexual activity; thus we did not divide the measured values by the total time. For the sexual focus index, SFI, Oregon-R-males performed 36% better than A30P *α*-syn males (Figure 1(a)). As can be seen from the results, *α*-syn A30P males performed less sexual activity (up to 50% reduction relative to Oregon-R activity) in all sexual parameters (Figure 1(a)).

For further comparison, all parameters of the six traits were summed up and normalized for each group. While each parameter is sufficient to distinguish between the two groups, we find the overall difference as a ratification of the results (Figure 1(b)). Since the six behavioral activities are not strictly independent, the summed score better represents the overall sexual behavior, and the result suggests that the A30P *α*-syn males were impaired in sexual focus, ability to follow females, and coordination (Figure 1(b)).

### 3.2. Climbing Assay

**Figure 2:**
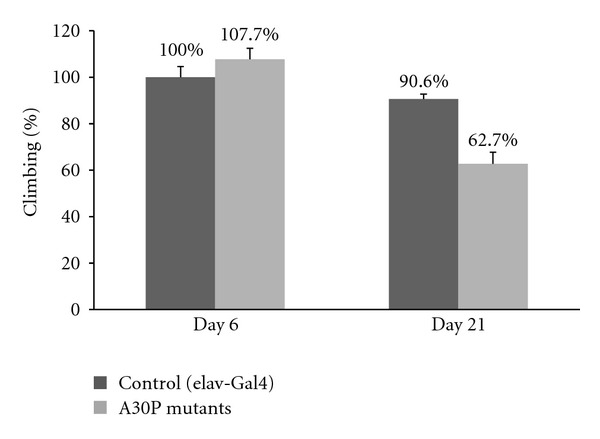
Climbing ability. Two classes of flies, each containing 5 tubes of 10 flies, were analyzed using the climbing assay. Control flies (shown in dark grey) are set as displaying 100% climbing ability. PD fly model presented in light grey. Results show the percent of flies which climbed along the test tube after 18 seconds.

The locomotion (climbing) assay, commonly used for assessing behavior of flies expressing amyloidogenic proteins in their brain [28–30], was used to assess the neural dysfunction caused to the flies due to expression of mutant A30P *α*-syn in the nervous system. We monitored the climbing ability of the flies at the age of 6 days, the age of flies tested in the courtship assay. As can be seen in Figure 2, no difference was detected between Oregon-R flies (control) and A30P *α*-syn flies as expected in this model. Additional measurement conducted at the age of 21 days revealed significant locomotor dysfunction of PD flies, reflecting 28% reduction in climbing ability (Figure 2). Our results did not indicate a significant difference between the two groups prior to 21 days (data not shown), as was also reported previously [12, 13].

### 3.3. Statistical Analysis

Based on the results obtained in the courtship assay, we used a data mining software to develop a set of rules (“if then” rules and “if and only if” rules) as a diagnostic tool. As each male fly represented an array of numbers, describing 8 parameters with regards to the 6 sexual activities, we established a two-dimensional matrix. Choosing health condition as the dependent variable, we used WizWhy 4.02 for the analysis as it (i) enables combined data sets analysis, (ii) relates to the whole set of data with no data modification or neglection, (iii) is less sensitive to overfitting (in small data sets), and (iv) is proven to reveal all “if then,” and a set of the necessary and sufficient conditions (“if and only if” rules) each with its significance level [31]. The software uses association rule learning algorithm to calculate the correlations that hold between the one independent variable, or combination of several parameters, the target function (the dependent variable), that is, to quantify the contribution of each of the 8 parameters to the decision whether a given fly is A30P *α*-syn (sick) or WT (healthy). The “if and only if” rule lists seven conditions (see Table 1). Our analysis revealed that if at least one of the seven conditions holds for a given fly, it was healthy with a probability of 82.1%. However, if none of the seven conditions holds, there was 82.1% probability that the fly carries *α*-syn A30P mutation (Table 1). Thus we were able to predict the health condition of 82.1% (46 out of 56 couples) of the flies. 10 flies (17.9%) did not follow this set of rules, leading to an improvement factor of 2.8 (0.5/0.179) in comparison to random prediction (Table 1).

## 4. Discussion

*Drosophila* has been used extensively as a model for human brain diseases, mainly due to the simplicity of the experiments along with the similarity to humans. *Drosophila* has a central nervous system containing orders of magnitude fewer neuronal and glial cells than in vertebrate central nervous systems, yet they share the same types of neurotransmitter systems such as GABA, glutamate, dopamine, serotonin, and acetylcholine, and they are able to perform complex behavior, including sexual displays, social behavior, and learning [32].

In this report, we present an alternative behavioral assay, employing courting pairs, for monitoring behavioral deficits in the *α*-syn A30P fly model and compare it to the well-established climbing assay. Courtship in *Drosophila* was studied and described in detail [20, 33–36], but to the best of our knowledge, this is the first time that it is characterized in a PD fly model. We examined five essential components of the male's courtship ritual and suggest one new activity, NSE, and its inverse, SFI. In all traits, PD male flies performed worse. Surprisingly, NSE was the most well-represented characteristics in the if-and-only-if rules, composing 4 out of the 7 derived conditions. This suggests that *α*-syn A30P mutant males are less responsive to females than are Oregon-R males. Further study is needed to explore the relationship between courtship and sexual focus in male PD model flies.

When compared to the well-established climbing assay, thoroughly reported as a convenient behavioral measure to determine neurological damage and aging in *Drosophila* flies [12, 13], courtship is a more complex behavior to assay. In courtship, the male must follow the female closely and engage in several coordinated behaviors, which is physically more demanding than simple climbing and which engages all the senses, as described previosly. Furthermore, the courtship abnormality is apparent at a much earlier age than the climbing deficiency. While at age 5–10 days the PD male flies court maximally, it takes approximately three weeks for the appearance of severe climbing phenotype in them (Figure 2). On the other hand, the climbing assay yields a binary score, pass or fail, whereas courtship must be evaluated quantitatively. Our results immediately suggest a follow-up experiment, evaluating behavior with the courtship assay at various ages post eclosion.

On balance, this behavioral assay provides a better evaluation of PD pathology dysfunction, and may allow assessment of dopaminergic dysfunction prior to loss of dopaminergic neurons, although the exact neuronal deficits underlying this behavioral phenotype need to be determined in follow-up studies. Interestingly, in the case of Fragile X, another brain disorder modeled in *Drosophila*, McBride et al. [37] demonstrated that lithium or mGluR antagonists could rescue several aspects of behavior including courtship impairments. This was followed by studies in the mouse model and now clinical studies in afflicted patients [38, 39]. Therefore, behavioral deficiencies during courtship in disease model flies can be an important and relevant assay for drug screening.

## Figures and Tables

**Table 1:** Data mining analysis. An “if and only if” rule, composed of six conditions, was concluded using WizWhy 4.02. Improvement factor of 2.8 (relative to random prediction) was observed.

*The following conditions explain when male fly is healthy:*
(i) Non-Sexual Encounters occurs 3 to 5 times (average = 4.40)
(ii) Orientation time is 294.00–349.51 (average 320.84)
& Total ritual time is 354.95–407.00 (average = 376.29)
(iii) attempted Copulations occur 3 times
(iv) Licking ig conducted 21 to 56 times (average = 34.50)
& Non-Sexual Encounters occur 6 to 17 times (average = 4.40)
(v) Non-Sexual Encounters occur 1 to 2 times (average = 100.40)
(iv) Vibration of wings last 0 to 77.00 seconds (average = 15.72)
& Non-Sexual Encounters occur 35 to 51 times (average = 43.43)
(iiv) Total ritual times is 516.58 to 519.91 (average = 517.94)
When *at least one* of the conditions holds, the probability that male fly *is* healthy equals *0.821 *
(23 out of 28 cases)
When *all* the conditions do not hold, the probability that male fly *is not* healthy equals *0.821* (23 out of 28 cases)
The total number of cases explained by the set of conditions: 46
The total number of cases in the data: 56
*Success rate: 0.821* (46/56)
Assuming that the primary probability for a male fly to be healthy equals 0.5 we obtain an
*Improvement Factor of 2.800* (0.500/0.179)
